# Simultaneous Color Contrast Increments with Complexity and Identity of the Target Stimulus

**DOI:** 10.3390/life15020257

**Published:** 2025-02-08

**Authors:** Paolo A. Grasso, Federico Tommasi, Rebecca Franconi, Elisabetta Baldanzi, Alessandro Farini, Massimo Gurioli

**Affiliations:** 1 Department of Physics and Astronomy, University of Florence, 50019 Florence, Italy; federico.tommasi@unifi.it (F.T.); rebecca.franconi@edu.unifi.it (R.F.); elisabetta.baldanzi@unifi.it (E.B.); alessandro.farini@unifi.it (A.F.); massimo.gurioli@unifi.it (M.G.); 2 National Research Council, National Institute of Optics, 50125 Florence, Italy

**Keywords:** simultaneous color contrast, structural complexity, identity

## Abstract

Simultaneous color contrast is a perceptual phenomenon in which a target stimulus appears to change its hue due to color induction from the surrounding background. In this study, we investigated whether this phenomenon is influenced by the structural complexity and identity of the stimuli used. In Experiment 1, we created two sets of stimuli varying in structural complexity and asked participants to perform a color-matching task on the achromatic target. Low-complexity targets consisted of simple squares, while high-complexity targets were stylized cars. The results showed that high-complexity stimuli triggered stronger color induction from the background and exhibited greater interindividual variation in perceived color saturation. Conversely, low-complexity stimuli were predominantly perceived as achromatic across all participants. In Experiment 2, we further explored whether these effects were influenced by differences in the stimuli’s topology and identity. Topological factors were controlled by ensuring similar organizations of stimulus elements across conditions, while the role of stimulus identity was examined by including a condition in which the high-complexity stimuli from Experiment 1 were presented in a scrambled arrangement, preventing recognition. The results demonstrated that color contrast increased with the complexity of the stimuli but also highlighted the role of identity, as the condition where the car was recognizable elicited the strongest color induction. We conclude that simultaneous color contrast is strengthened by factors that pertain to both the complexity of the stimuli used and their identity.

## 1. Introduction

Colors are an integral part of our daily lives, playing a crucial role in the rapid detection and categorization of objects and significantly influencing perceptual judgments [1,2,3,4]. Our experience of color is based on the ability to discriminate wavelengths of light reflected by surfaces. This ability is mediated by the activation of cone photoreceptors, which transduce light into neural signals. These signals are then combined to produce opponent responses that are transmitted to the brain [5,6].

However, this description does not fully account for a range of perceptual phenomena related to color appearance. One of the most striking examples is simultaneous color contrast, where a target appears to change its hue due to interactions with its surrounding background [7,8]. The origins of this phenomenon are still debated, but numerous studies have sought to understand variations in its strength under different methodological conditions [9]. For example, the vividness of chromatic induction has been shown to depend on the saturation of the background, with more saturated backgrounds inducing more intense color experiences [10]. Furthermore, the magnitude of simultaneous color contrast has been found to decrease with increasing target-surround contrast. A low-contrast target in a uniform background appears more saturated than the same target in a variegated background, although this difference diminishes as the target’s contrast increases [11,12]. Moreover, some authors proposed that simultaneous contrast involves separate “fast” and “slow” mechanisms, with stronger induction effects for fast mechanisms than slow ones [13,14,15]. In line with this, recent research has suggested that simultaneous color contrast may be linked to the perception of transparency, which tends to be stronger for low-contrast stimuli [16].

Despite these findings, it remains unclear whether higher-order factors such as stimulus identity and structural complexity influence the phenomenon. This study aimed to address this question by creating purpose-built stimuli designed to induce chromatic effects from the background while differing in complexity and identity. The stimuli were carefully balanced for low-level visual properties but systematically varied in terms of structural complexity and the presence or absence of recognizable identity.

## 2. Methods

### 2.1. Participants

Thirty participants took part in Experiment 1 (mean age: 22.7 years, SD: 2.56 years; 18 females; age range: 18–30 years) and thirteen participants took part in Experiment 2 (mean age: 22 years, SD: 1.51 years; 9 females; age range: 20–25 years). All participants had normal or corrected-to-normal visual acuity and normal color vision as assessed by the Ishihara Test.

### 2.2. Assessment of Simultaneous Color Contrast

**Figure 1:**
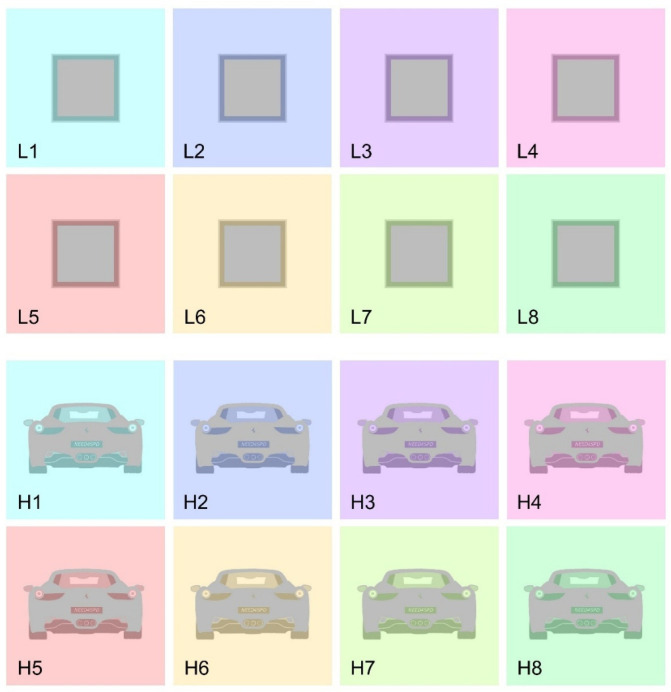
Stimuli employed in Experiment 1. L1 to L8 depict stimuli employed in the low-complexity condition, while H1 to H8 depict stimuli employed in the high-complexity condition.

**Figure 2:**
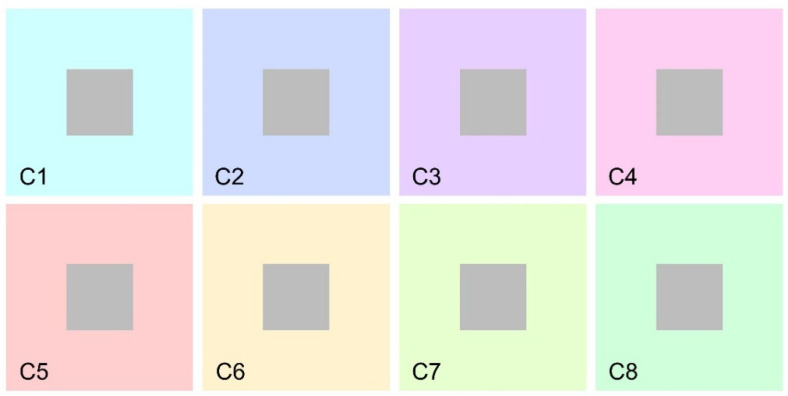
C1 to C8 depict stimuli employed in the control condition in Experiment 1.

**Figure 3:**
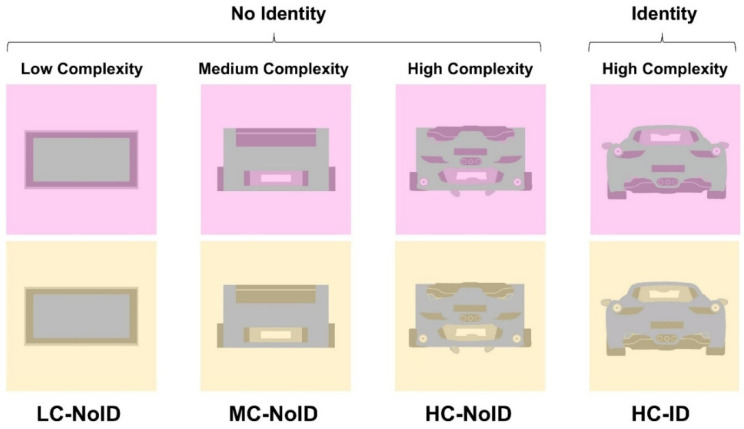
Stimuli employed in Experiment 2. Condition LC-NoID depicted stimuli with a low-complexity arrangement of elements and lack of identity. Condition MC-NoID depicted stimuli with a medium-complexity arrangement and lack of identity. Condition HC-NoID depicted stimuli with a high-complexity arrangement and lack of identity. Condition HC-ID depicted stimuli with a high-complexity arrangement and a clear identity.

The magnitude of simultaneous color contrast was measured using a matching procedure. Participants were instructed to match the perceived color of a series of achromatic stimuli (hue: 0°; saturation: 0; value: 0.74) embedded within various chromatic backgrounds (see Figure 1, Figure 2 and Figure 3 for examples of stimuli employed).

#### 2.2.1. Experiment 1

In Experiment 1, we designed two conditions to investigate the role of structural complexity in the magnitude of color induction. Structural complexity was broadly defined as the level of detail or intricacy within an image [17] with perceived complexity positively correlating with the variety present in a picture [18]. In the first condition, structural complexity was kept “low” (low-complexity condition), where the stimulus to be matched was a simple gray square surrounded by a dark frame and a light frame. In the second condition, structural complexity was relatively “high” (high-complexity condition), where the stimulus to be matched was the body of a car, featuring sketch-like details such as rear-view mirrors, a rear window, rear lights, and a license plate. Importantly, the two stimuli (i.e., the square and the car) were comparable in terms of low-level visual characteristics. The car’s body shared the same gray tone and area as the square, while the car’s details (e.g., rear-view mirrors, rear window, and rear lights) matched the cumulative area and colors of the two frames surrounding the square in the low-complexity condition. Thus, the primary difference between the two conditions was the level of structural complexity provided (Figure 1).

However, it is worth noting that in the low-complexity condition, the target stimulus was consistently surrounded by a dark frame, whereas in the high-complexity condition, the car’s contours directly bordered the background in most areas. To control the potential influence of this difference, we included a control condition in which the stimuli from the low-complexity condition were presented directly bordering the background (Figure 2). In each condition of Experiment 1, participants were shown eight distinct images, each with a different background hue designed to induce varying levels of color in the target stimulus (see Figure 1 and Figure 2).

#### 2.2.2. Experiment 2

However, we acknowledge that the stimuli used in the two conditions (i.e., low and high complexity) also differed in several other respects. For example, high-complexity stimuli were associated with an “identity”, the recognition of the object as a sketched car, while the low-complexity stimuli did not evoke any familiar object beyond a simple geometric shape. Additionally, the high-complexity stimuli included colored details, not only around the contours of the car but also within its body. To control the potential effects of these differences, in Experiment 2, we explored simultaneous color contrast in four new conditions, with a different set of participants.

The first condition mirrored the low-complexity condition from Experiment 1, featuring a simple low-complexity arrangement and the absence of any identifiable stimulus (LC-NoID). The second condition involved a medium-complexity arrangement, incorporating colored details within the achromatic surface but still lacking identity (MC-NoID). The third condition involved a scrambled version of the car image (from the high-complexity condition in Experiment 1), creating a high-complexity arrangement without identity (HC-NoID). Finally, the fourth condition replicated the high-complexity condition from Experiment 1, featuring both high complexity and an identifiable stimulus (HC-ID). These manipulations enabled a thorough examination of the effects of complexity, topological factors related to color arrangements, and identity. Consistent with Experiment 1, the cumulative area covered by the different colors was comparable across all four conditions.

In each condition of Experiment 2, participants were presented with two distinct images, each with a different background hue designed to induce a varying color experience in the target stimulus (see Figure 3).

### 2.3. General Procedure and Equipment Details

**Figure 4:**
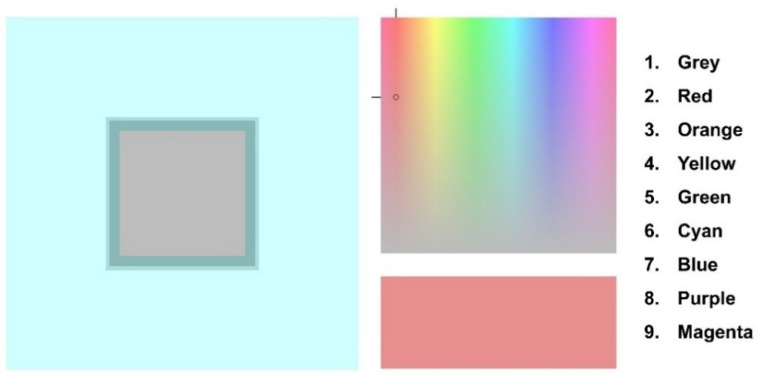
An example of a trial. Participants navigated a cursor (black circle) through a fixed-luminance color-space diagram to find the combination of hue and saturation that better matched the individual perception of the color of the target stimulus (color matching). The rectangle below changed its color according to the position of the cursor. Afterwards, they had to classify their perceptual experience by selecting the closest match within a set of predetermined color names (color classification).

Stimuli were presented on a linearized and calibrated LED monitor. Participants had to navigate a cursor on a fixed-luminance color-space diagram to change the color of a nearby rectangle (see Figure 4) and were instructed to select the combination of hue (horizontal shift) and saturation (vertical shift) that better matched the target stimulus (color matching). Afterwards, they were asked to classify their perceptual experience by selecting the closest match within a predetermined set of color names (color classification). Each image was displayed three times, and the order of stimuli was randomized.

This procedure generated an RGB triplet corresponding to the response provided to each image. Each RGB triplet was transformed into HSV coordinates using the in-built Matlab function rgb2hsv [19] and, afterwards, hue and saturation values corresponding to the same image were averaged across different trials. The experiment was run using a custom script built in Matlab 2021b (The Mathworks, Inc., Natick, MA, USA) with stimuli being presented using PsychToolbox 3 routines [20].

The calibration and characterization of the display (24 inches, refresh rate: 60 Hz, 1920 × 1080-pixel resolution) were made using the approach described by Westland and collaborators [21]. We used a Konica Minolta CS-1000 (Chiyoda, Tokyo, Japan) spectrophotometer measuring spectra and colorimetric quantities. With this procedure, we were able to link RGB values with CIE 1931 XYZ values using gogvals and M retrievable in Table 1 and Table 2.

### 2.4. Statistical Analyses

Analyses were performed using JASP 0.14.1.0 (University of Amsterdam, Amsterdam, The Netherlands). Repeated measures ANOVAs and paired sample *t*-tests were used to compare different experimental conditions. To compensate for violations of sphericity, Greenhouse–Geisser corrections were applied whenever appropriate [22] and corrected *p*-values (but uncorrected degrees of freedom) are reported. Post hoc comparisons were performed using Bonferroni correction.

## 3. Results

### 3.1. Experiment 1

The first experiment was designed to test the role played by complexity, defined as the level of detail or intricacy within an image [17]. To contrast differences in saturation responses between low and high complexity from Experiment 1 conditions, we performed a 2 × 8 within-factor ANOVA with complexity (low and high) and color (1 to 8) as factors. Results revealed a highly significant main effect of complexity (*F*_(1,29)_ = 32.41; *p* < 0.001; *ƞ_p_*^2^ = 0.52) explained by higher saturation responses in the high-complexity condition (mean: 0.14) compared to the low-complexity condition (mean: 0.03; *p* < 0.001). Also, a main effect of color emerged (*F*_(7,203)_ = 15.85; *p* < 0.001; *ƞ_p_*^2^ = 0.35) suggesting that the eight background colors elicited different amounts of simultaneous color contrast regardless of complexity. Finally, an interaction between complexity and color emerged (*F*_(7,203)_ = 10.57; *p* < 0.001; *ƞ_p_*^2^ = 0.26), which has to be mainly attributable to differences in the amount of chromatic induction between stimuli selectively evident in the high-complexity condition, rather than representing a lack of difference between low and high complexity for some specific set of stimuli. Indeed, all direct comparisons between low- and high-complexity conditions among the same set of stimuli (e.g., L1 vs. H1, L2 vs. H2, etc.) were highly significant (all *p <* 0.001).

**Figure 5:**
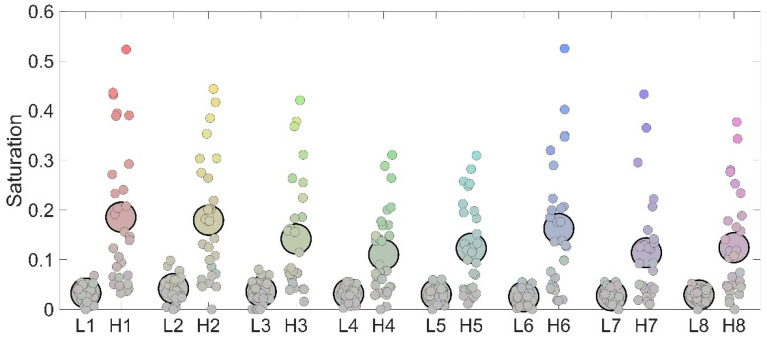
Individual (small dots) and average (large dots) responses to the eight stimuli employed in terms of saturation. The color assigned represents the combination of hue and saturation that each participant chose to best represent the perceived color of the target stimulus. For each image, left-sided dots represent responses in the low-complexity condition while right-sided dots represent responses in the high-complexity condition.

Importantly, the increased saturation responses for the high-complexity condition were evident across almost all the participants as 93.3% of participants reported larger saturation in the high-complexity condition compared to the low-complexity condition. Furthermore, Figure 5 also shows that the two conditions were characterized by a clear difference in terms of variability of responses as in the high-complexity condition, saturation responses varied a lot, spanning from close to 0 to more than 0.5, while in the low complexity, saturation responses were condensed in the range between 0 and 0.08.

**Figure 6:**
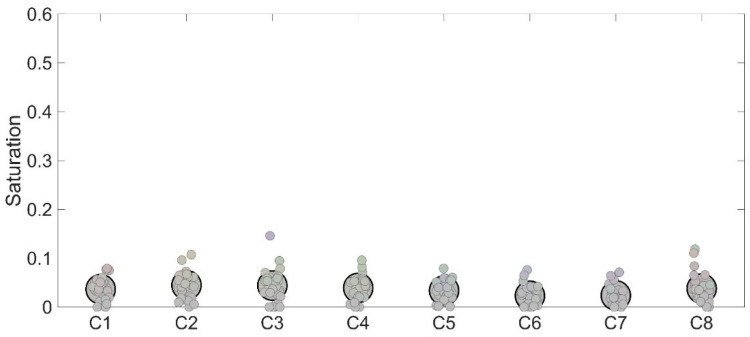
Individual (small dots) and average (large dots) responses to the eight stimuli employed in the control condition in terms of saturation. The color assigned represents the combination of hue and saturation that each participant chose to best represent the perceived color of the target stimulus.

However, one may be convinced that the difference reported between high- and low-complexity conditions could be due to the fact that in the low-complexity condition, a dark frame was continuously interposed between the target stimulus and the background and this was not the same in the high-complexity condition where the body of the car was mostly directly bordering the background. To control the potential role played by this difference, we assessed simultaneous color contrast in an additional control condition in which no frame was interposed between the central gray square and the background. Results made evident that this condition produced results in close agreement with those reported in the low-complexity condition, thus ruling out the possibility that the weak color contrast found in such a condition could be ascribed to the larger amount of interposed dark regions between the target and the background (Figure 6).

These results made evident that the high-complexity condition led to a much larger chromatic induction compared to both the low-complexity and the control condition. This was also evident from the classification task. The percentage of times participants perceived the stimulus as achromatic drastically dropped in the high-complexity condition (Figure 8A). This was evident across all eight stimuli employed, although some variations were present as, for instance, stimuli 3 and 4 had a greater chance to be classified as gray even in the high-complexity condition.

### 3.2. Experiment 2

In the second experiment, we aimed at stepping further in the direction of results obtained in our first experiment. Indeed, data obtained from Experiment 1 cannot rule out that the increase in the high-complexity condition could be due to other differences between the two conditions such as the presence of colored elements inside the gray area in the high-complexity condition but not in the low-complexity one, or the fact that the high-complexity condition was characterized by a clear stimulus identity (i.e., being a car). Experiment 2 aimed at disentangling such points. Participants were presented with four types of stimuli. The first was identical to the one used in the low-complexity condition of Experiment 1, featuring low complexity and no identifiable shape (LC-NoID). The second had a medium level of complexity, with additional elements distributed within the target stimulus but no identity (MC-NoID). The third had high complexity, created by scrambling elements from the high-complexity stimuli in Experiment 1 to prevent recognition (HC-NoID). The fourth category consisted of the original high-complexity stimuli from Experiment 1, which maintained both high complexity and a recognizable identity (HC-ID).

**Figure 7:**
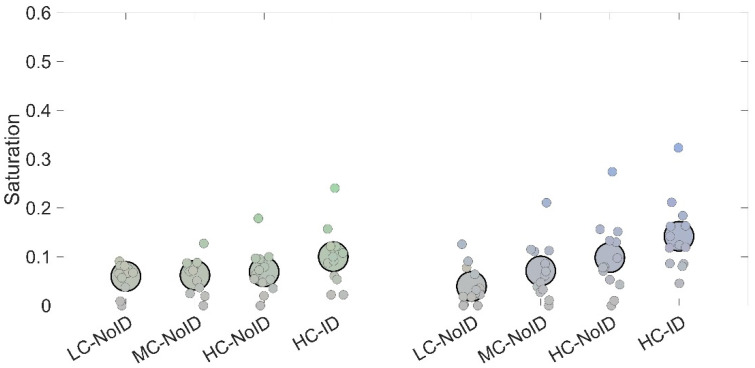
Saturation estimates obtained in the four conditions employed in Experiment 2. The left side depicts stimuli with a magenta background; right-side stimuli had a yellow background. Small dots depict individual while large dots depict average responses.

A 4 × 2 within-factor ANOVA with condition (LC-NoID, MD-NoID, HC-NoID, HC-ID) and color (two levels) as factors was performed. Results revealed a main effect of condition (*F*_(3,36)_ = 21.16; *p* < 0.001; *ƞ_p_*^2^ = 0.64), which was explained by the HC-ID condition producing significantly higher saturation estimates compared to each of the other three conditions (all *p* < 0.002). Also, a condition x color interaction emerged (*F*_(3,36)_ = 6.43; *p* = 0.001; *ƞ_p_*^2^ = 0.35), which was mainly explained by a differential increase in saturation as a function of complexity between the two colors used (see Figure 7). More specifically, the HC-ID condition showed higher saturation estimates compared to all the other conditions for stimuli with a yellow background (Figure 7, right panel), while it showed higher saturation estimates compared to LC-NoID and MC-NoID for stimuli with a magenta background (Figure 7, left panel).

**Figure 8:**
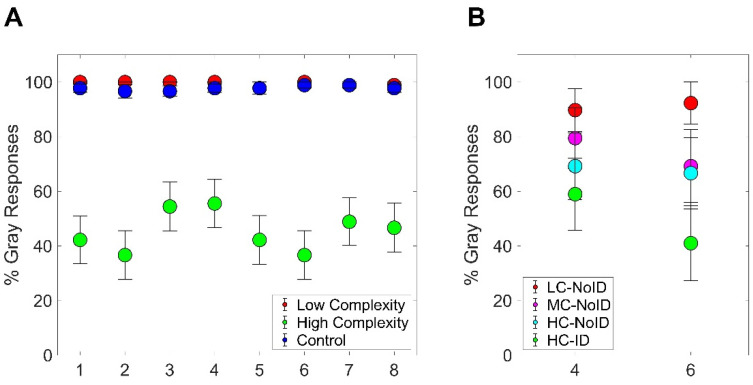
(**A**) Percentages of gray responses obtained in the low-complexity, high-complexity, and control conditions of Experiment 1 for each of the eight stimuli employed. Error bars represent standard errors of the mean. (**B**): Percentages of gray responses obtained in the four conditions employed in Experiment 2 (LC-NoID, MC-NoID, HC-NoID, HC-ID) for each of the two stimuli employed. Error bars represent standard errors of the mean.

Taken together, the results from Experiment 2 confirm that a complex arrangement of elements within a target stimulus leads to a significantly stronger chromatic induction. Furthermore, these findings highlight the role of stimulus identity in generating color contrast, as the condition using a recognizable stimulus produced the largest chromatic induction. This effect was also evident in the classification task, where the likelihood of participants perceiving the stimulus as achromatic decreased as stimulus complexity and identity increased (Figure 8B).

## 4. Discussion

In this study, we further investigated the factors influencing simultaneous color contrast, a phenomenon in which a target stimulus appears to shift its hue as a consequence of the background in which it is embedded [23]. Specifically, the target stimulus tends to shift its hue toward the complementary color of the background. This “illusory” perceptual experience demonstrates that color perception is a global process, influenced by high-order mechanisms. Here, we examined whether factors such as the structural complexity and/or identity of a stimulus play a role in this phenomenon. Participants were asked to match the color of a central achromatic target stimulus embedded in various colored backgrounds by moving a cursor on a fixed-luminance color-space diagram. In Experiment 1, stimuli varied in complexity: low-complexity stimuli consisted of simple squares, while high-complexity stimuli were stylized cars. Our findings support the notion that complexity enhances chromatic induction, as strong chromatic effects were observed exclusively for the high-complexity stimuli. To control low-level differences between the two categories, both low- and high-complexity stimuli were designed to display identical colors, with the cumulative area covered by each color being roughly equivalent. Thus, the primary distinction between the two categories was the degree of structural complexity. Results revealed a pronounced simultaneous color contrast effect for high-complexity stimuli, whereas low-complexity stimuli induced only a faint color hue in the achromatic target.

Additionally, the high-complexity condition exhibited greater interindividual variability in saturation responses compared to the low-complexity condition, in which responses were more uniform. However, one might argue that differences in contour transitions between the target and background could have contributed to these results, as previous research has shown that contours influence the strength of simultaneous color contrast [24]. To address this concern, we included a condition with gray squares embedded in colored backgrounds but without any surrounding frame. The results for this condition closely resembled those of the low-complexity condition, suggesting that contour differences were unlikely to account for the observed effects.

Since the two stimulus categories also differed in other aspects, such as the topographical arrangement of colored elements and stimulus identity, Experiment 2 was designed to control for these confounds. In this experiment, we introduced two additional conditions to disentangle the roles of stimulus identity and topological complexity. Results showed that saturation estimates increased with stimulus complexity. Notably, the condition featuring a recognizable car (HC-ID) elicited significantly stronger saturation estimates compared to both simpler stimuli and the condition with a scrambled car (HC-NoID). This finding suggests that the stronger saturation effects observed in the high-complexity condition of Experiment 1 were not solely driven by structural complexity but also by the identity of the stimulus. Our results align with previous evidence on the relationship between transparency perception and simultaneous color contrast, which suggests that a more realistic perception of transparency enhances color contrast [16]. One possibility is that increasing the complexity and topological distribution of colored elements within the target stimulus helps observers estimate the filter’s transmittance, resulting in a more vivid color illusion. A similar effect may apply to identity: when the target is a recognizable object, it may strengthen the impression of viewing a real image through a transparent filter.

In summary, our findings suggest that the structural components of an image contribute to the strength of chromatic induction. Specifically, more “realistic” images appear to elicit a more vivid illusion of color. We speculate that this may be due to higher-order cognitive processes related to the identity of the stimulus, which are bound to its structural complexity.

## Figures and Tables

**Table 1:** Gamma and gain values for the three channels’ RGB for the display used in the task.

Gogvals	
2.24	0.91
2.25	0.92
2.21	0.92

**Table 2:** Transformation matrix from RGB to XYZ for the display used in the task.

M		
82.8	37.7	29.5
37.8	102.9	13.8
2.3	16.3	150.5

## Data Availability

The raw data were deposited in the Zenodo repository and are publicly available at the following link: https://zenodo.org/records/14541019 (accessed on 3 February 2025).
